# Rethinking the rise of early-onset gastrointestinal cancers: a call to action

**DOI:** 10.1093/jncics/pkaf002

**Published:** 2025-01-15

**Authors:** Benjamin A Weinberg, Caitlin C Murphy, David R Freyer, K Leigh Greathouse, Jan K Blancato, Elena M Stoffel, Julia L Drewes, Anne Blaes, John M Salsman, Y Nancy You, Hannah Arem, Reetu Mukherji, Priyanka Kanth, Xin Hu, Anne Fabrizio, Marion L Hartley, Marios Giannakis, John L Marshall

**Affiliations:** Ruesch Center for the Cure of Gastrointestinal Cancers, Lombardi Comprehensive Cancer Center, Georgetown University Medical Center, Washington, DC 20007, United States; Department of Health Promotion & Behavioral Sciences, UTHealth Houston School of Public Health, Houston, TX 77030, United States; Departments of Pediatrics, Medicine, and Population & Public Health Sciences, Keck School of Medicine, University of Southern California, Los Angeles, CA 90032, United States; USC Norris Comprehensive Cancer Center and Children’s Hospital Los Angeles, Los Angeles, CA 90089, United States; Department of Human Sciences and Design, Baylor University, Waco, TX 76706, United States; Department of Oncology, Georgetown University, Washington, DC 20007, United States; Department of Internal Medicine, University of Michigan, Rogel Cancer Center, Ann Arbor, MI 48109, United States; Department of Medicine, Division of Infectious Diseases, Johns Hopkins University School of Medicine, Baltimore, MD 21287, United States; Masonic Cancer Center, University of Minnesota, Minneapolis, MN 55455, United States; Wake Forest University School of Medicine & Atrium Health Wake Forest Baptist Comprehensive Cancer Center, Winston Salem, NC 27157, United States; Department of Colon and Rectal Surgery, University of Texas MD Anderson Cancer Center, Houston, TX 78701, United States; Healthcare Delivery Research, MedStar Health Research Institute, Washington, DC 20010, United States; Ruesch Center for the Cure of Gastrointestinal Cancers, Lombardi Comprehensive Cancer Center, Georgetown University Medical Center, Washington, DC 20007, United States; Division of Gastroenterology, MedStar Georgetown University Hospital, Lombardi Comprehensive Cancer Center, Washington, DC 20007, United States; Gangarosa Department of Environmental Health, Emory University Rollins School of Public Health, Atlanta, GA 30329, United States; Division of Colon & Rectal Surgery, MedStar Georgetown University Hospital, Washington, DC 20007, United States; Ruesch Center for the Cure of Gastrointestinal Cancers, Lombardi Comprehensive Cancer Center, Georgetown University Medical Center, Washington, DC 20007, United States; Dana-Farber Cancer Institute and Harvard Medical School, Boston, MA 02215, United States; Ruesch Center for the Cure of Gastrointestinal Cancers, Lombardi Comprehensive Cancer Center, Georgetown University Medical Center, Washington, DC 20007, United States

## Abstract

Since the early 1990s, there has been a dramatic rise in gastrointestinal cancers diagnosed in patients under age 50 for reasons that remain poorly understood. The most significant change has been the increase in incidence rates of early-onset colorectal cancer, especially rates of left-sided colon and rectal cancers. Increases in gastric, pancreatic, and other gastrointestinal cancer diagnoses have further contributed to this trend. We formed a multidisciplinary Think Tank to develop a strategic, coordinated approach to studying early-onset gastrointestinal cancers. This area of research is challenging given multifactorial etiologies. We focused on epidemiology and the environment, the microbiome, and survivorship as key pillars to structure a research framework. We advocate a comprehensive strategy to (1) use existing biospecimens, especially those collected longitudinally, with correlation to exposures (the *exposome*); (2) standardize microbiome specimen collection and analyses of blood, tissue, and stool specimens to minimize contamination and biases; (3) prioritize mechanistic studies to evaluate findings from biomarker studies; and (4) explore the unique survivorship needs of this young population. These recommendations build upon prior efforts with the goal of streamlining research into this important field of study while minimizing redundant efforts. We hope that our findings serve as a clarion call to motivate others to discover why young individuals are being diagnosed with gastrointestinal cancers at such an alarming rate and how to best support those who have been diagnosed.

## Introduction

The rise in early-onset gastrointestinal cancers (EOGICs) is an emerging clinical and public health problem.^1^^,^^2^ Health-care providers are seeing an unmistakable, steady increase in young individuals younger than age 50 seeking care for EOGIC. Despite a growing body of literature, particularly concerning early-onset colorectal cancer (EOCRC), we still do not have a firm understanding of the etiology of EOGIC. Research programs studying early-life exposures, tumor genomics, epigenomics, and the microbiome have only partially helped elucidate potential causes for EOGIC. We brought together experts across disciplines to formulate a unified strategic framework to move the field of EOGIC forward.

Our multidisciplinary approach was unique for multiple reasons. First, we intentionally broadened our focus beyond EOCRC and discussed other EOGICs, including gastric, esophageal, pancreatic, and hepatobiliary cancers. Second, we incorporated experts in cancer survivorship. The needs of patients surviving EOGICs represent specific challenges, as they are often in the early stages of careers and child-rearing and live longer with long-term toxicities of treatment than older patients. Third, we gathered experts in diverse fields of epidemiology, gastroenterology, oncology, survivorship, and microbiome to offer their opinions about how they would prioritize efforts to study EOGIC.

This EOGIC Think Tank was convened by the Ruesch Center for the Cure of Gastrointestinal Cancers. The mission of the Ruesch Center is to combine expertise in molecular medicine, translational research, and a patient-centered philosophy to realize the dream of individualized curative therapies through research, care, and advocacy. The Think Tank epitomizes our collaborative desire to prevent, treat, and support those with EOGIC, and this article serves as a call to action to better motivate and position our research strategy to accomplish this goal.

## Methods

The Think Tank on Research Needs in EOGICs convened national experts with active research and clinical programs related to EOGICs to serve as thought leaders for review and discussion of the following chosen areas: epidemiology and the environment, microbiome, and survivorship. Breakout groups, each chaired by an invited expert, comprised population health scientists, molecular and cell biologists, oncologists, gastroenterologists, hepatologists, surgeons, and cancer survivors from the Ruesch Center and external institutions. A scribe was assigned to each working group to capture comments. Summary statements and recommendations were abstracted from the working group notes and distributed back to the group for edits and further comments.

The Think Tank was held in Washington, DC, on the afternoon before the 2-day Ruesch Annual Symposium on GI Cancer. The Ruesch Symposium draws oncologists, geneticists, scientists, patients, and their caregivers for updates on the state of the science of gastrointestinal (GI) cancers. Selected Think Tank participants presented their findings to that larger audience the following day as a critical part of the symposium. During the Think Tank afternoon sessions, key speakers presented a 20-minute review of the current literature and their research findings on the 3 topic areas, followed by question and answer sessions. This article summarizes our findings from the 3 topic areas as well as action items to realize the goal of understanding, preventing, treating, and supporting patients with EOGICs.

## Epidemiology and the exposome

**Figure 1.**
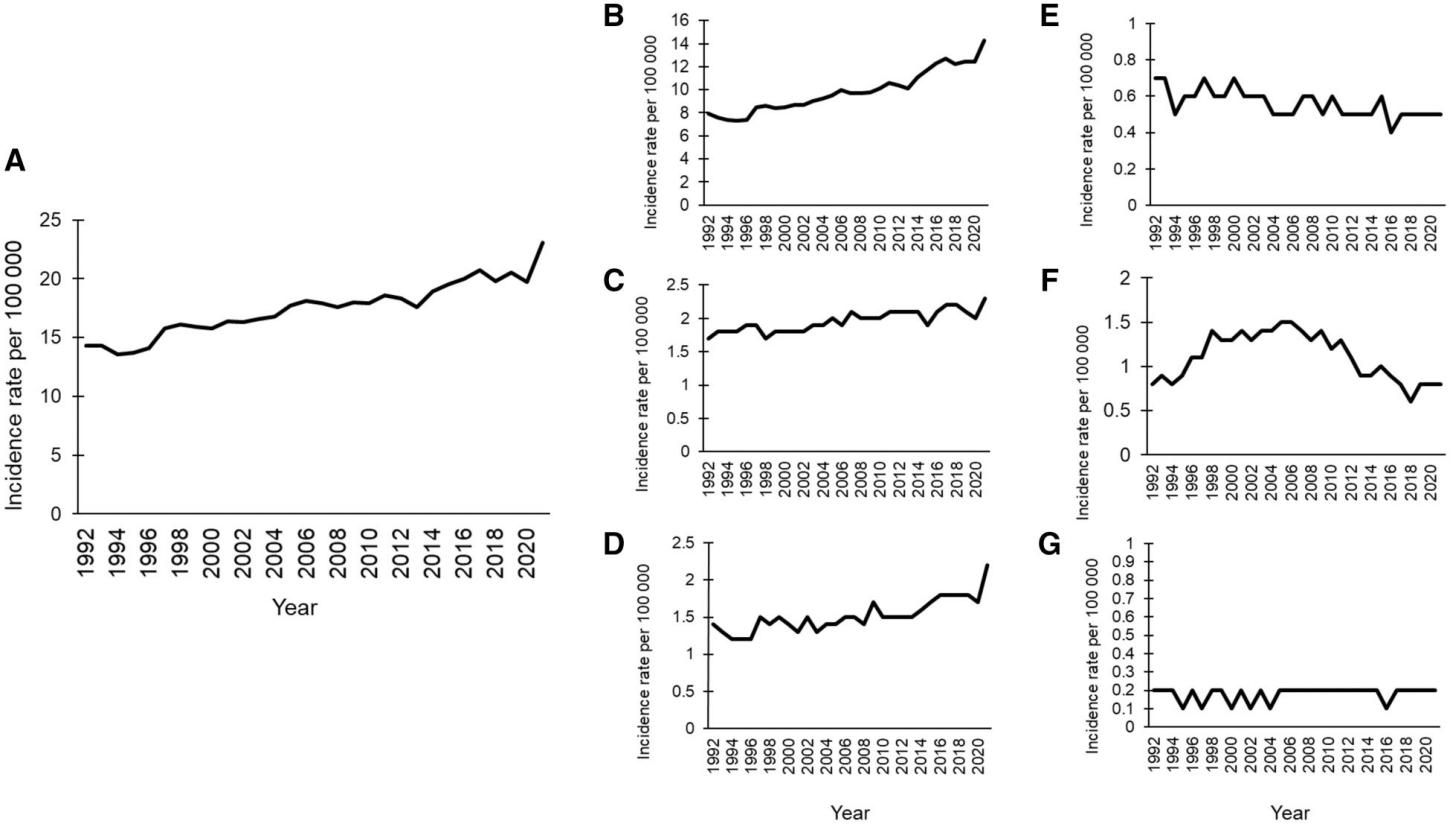
Increase in incidence rates of EOGIC, 1992-2021 (SEER Data), prepared by C.C.M. from the Surveillance, Epidemiology, and End Results (SEER) Program SEER*Stat Database: **A**) all GI cancers, **B**) colorectal cancer (CRC), **C**) gastric cancer, **D**) pancreatic cancer, **E**) esophageal cancer, **F**) hepatocellular carcinoma (HCC), and **G**) gallbladder cancer.^3^ Abbreviation: EOGIC = early-onset gastrointestinal cancer.

Incidence rates of EOGIC have increased since the early 1990s. We carried out our own analyses of SEER data (C.C.M.), and findings are depicted in Figure 1.^3^ Below, we outline 4 patterns in epidemiologic data that offer clues for understanding the etiology behind this increase.

First, the magnitude of absolute and relative increases in incidence rates varies by cancer type, suggesting that both shared and distinct risk factors are at play. Rates of EOCRC have driven the overall increase in EOGIC, increasing from 7.9 per 100 000 individuals in 1992 to 14.3 per 100 000 in 2021, an 81% relative increase.^3^ There have been smaller but still meaningful increases in early-onset gastric cancer (from 1.7 to 2.3 per 100 000, a 34% relative increase) and early-onset pancreas cancer (from 1.4 to 2.2 per 100 000, a 59% relative increase). In contrast, rates of hepatocellular carcinoma are decreasing among younger adults,^4^ partly due to the availability of direct-acting antivirals and subsequent decreases in chronic hepatitis C virus infection. Rates of early-onset esophageal (about 0.5 per 100 000) and gallbladder (about 0.2 per 100 000) cancers remain low and have not appreciably increased over time. It is worth noting that gastric cancer is the only cancer type for which the proportion of young adults with metastatic disease is greater than either middle-aged or older adults.^5^^,^^6^

Second, increasing incidence rates of EOGIC also vary by race and ethnicity, particularly for colorectal, gastric, and pancreas cancers. For example, incidence rates of EOCRC have increased across all racial and ethnic groups, but the increase has been more pronounced among non-Hispanic White persons. Rates in this group increased from 7.6 per 100 000 in 1992 to 15.2 per 100 000 in 2021, narrowing the previously noted gap in rates between non-Hispanic White and Black persons.^3^ Rates of EOCRC are also rapidly increasing among Hispanic persons (from 6.3 to 12.1 per 100 000), approaching rates observed in non-Hispanic White and Black persons. Conversely, rates of early-onset gastric cancer have either decreased or remained stagnant for all racial and ethnic groups except Hispanic persons, among whom rates increased from 2.8 per 100 000 in 1992 to 4.0 per 100 000 in 2021, now highest among all groups. One hypothesized reason for this rise is the increasing prevalence of Helicobacter *pylori* infection in young Hispanic populations, which is higher than in other races and older adults.^7^^,^^8^ Rates of early-onset pancreas cancer have also increased most rapidly among Hispanic persons (from 0.9 to 2.4 per 100 000) and to a lesser extent among non-Hispanic White persons (from 1.2 to 2.1 per 100 000). Racial and ethnic differences in the distribution of risk factors (eg, prevalence of diabetes) are highest among Hispanic persons^9^ and may contribute to these patterns and point to mechanisms underlying increases in EOGIC.

Third, rates of EOGIC have increased across successive birth cohorts, particularly among those born in and after the 1960s, such as Generation X and millennials, and the examination of early-life exposures in EOGIC patients appears to be important.^10-12^ These increasing rates are most notable for EOCRC^13^ but are also evident for early-onset gastric and pancreas cancers. For example, compared with persons born in 1955-1959, EOCRC rates are 1.30 times and 2.62 times higher for persons born in 1970-1974 and 1990-1994, respectively. Similarly, rates of early-onset gastric cancer are 1.35 times higher, and rates of early-onset pancreas cancer are 1.40 times higher among persons born in 1980-1984 compared with those born in 1955-1959. These “birth cohort effects” are common for many health outcomes and often coincide with population shifts in exposure to risk factors, particularly during early life. Birth cohorts age together, sharing exposures such as historical events, social experiences, and behavioral and environmental risk factors. Identifying exposures that have become more prevalent among higher risk birth cohorts may provide new opportunities for prevention and a better understanding of etiology.

Fourth, germline genetics cannot fully explain increasing incidence rates. Rates have increased more rapidly than expected, possibly due to changes in population genetics, and it is unlikely that the penetrance of hereditary cancer syndromes (eg, Lynch syndrome) has significantly increased over time. For example, more than 80% of persons with EOCRC do not have a germline mutation.^14-16^ Similar findings have been reported for early-onset gastric^17^ and pancreas^18^ cancers. Colorectal cancer (CRC) polygenic risk scores can point to common germline variants associated with EOCRC, especially when combined with environmental and lifestyle risk factors,^19^ as can EOCRC-specific genome-wide association studies and Mendelian Randomization analyses.^20^

Collectively, these patterns in epidemiologic data suggest that the increasing incidence rates of EOGIC are likely due to environmental exposures, which are globally termed the *exposome*. It is also likely that the exposome interacts with the genome, and therefore, gene × environment (G × E) interactions play a role. The concept of the exposome has evolved over the past 2 decades. It is now recognized as the cumulative impact of environmental exposures and associated biological responses across the life course.^21-23^ Increasing rates of EOGIC may be due to changes in the exposome, including increased exposure to pesticides, pollutants, and other environmental chemicals; changes in food composition and additives; changes in drugs and drug use; changes in lifestyle behaviors; climate change; and increases in the distribution and abundance of cancer-relevant viruses, bacteria, and fungi coupled with a potential loss of beneficial microbes.

The vast number and magnitude of changes to the exposome require new approaches. Simply put, “one exposure, one disease” models are insufficient for understanding complex diseases like EOGIC.^24^ Think Tank participants made several recommendations for studying etiology and addressing increasing rates, summarized below. These recommendations can generate hypotheses from observational and prospective cohort studies that can be tested further in animal and in vitro models.

**Figure 2.**
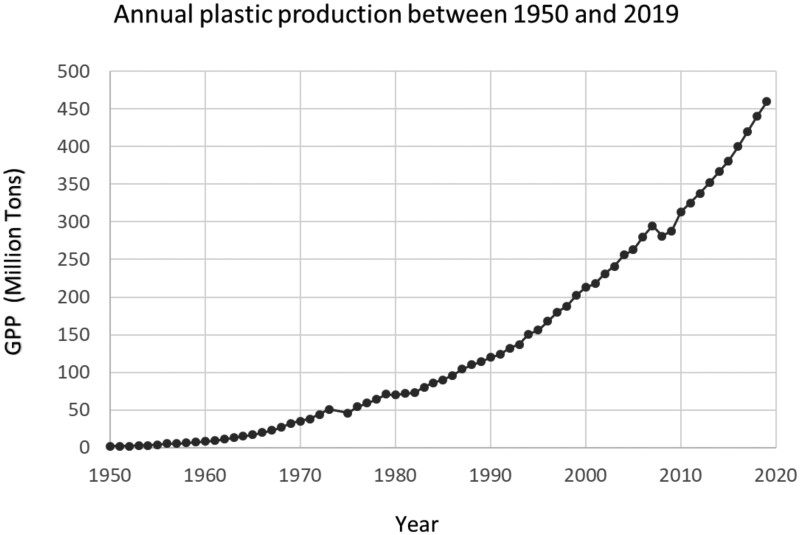
Global plastic production (GPP) from 1950 to 2015: the annual production of polymer resin and fibers. Ritchie, Hannah, and Max Roser. “Plastic pollution.” Our World in Data (2018).^26^^,^^33^

- Longitudinal studies starting in childhood, infancy, and even pregnancy, although ambitious, can provide additional insight into changes in the exposome and the impact of these lifetime changes on the risk of EOGIC. These studies will require the collaboration of adult and pediatric researchers and the standardization of assays and questionnaires across populations and time.
- Existing biospecimens, including cord blood, newborn blood spots, and prediagnostic specimens collected for unrelated purposes, can be leveraged for analyses; many of these biospecimens also align with etiologically relevant windows of exposure, such as during embryological and early childhood development.^22^ Alternatively, patients newly diagnosed with EOGIC may contribute biospecimens (eg, fresh tissue, blood, stool) for deep phenotyping. The Think Tank discussed the creation of a registry for EOGIC, similar to the Count Me In initiative (https://joincountmein.org), in which individuals can share their samples and clinical information and input their own exposure and other data.
- High-resolution mass spectrometry of biospecimens, such as blood, tissue, and stool samples, can identify environmental exposures associated with EOGIC, including previously unsuspected risk factors. Mass spectrometry methods have expanded our knowledge of the largely undefined exposome and identified new risk factors for disease, such as the microbiome-derived metabolite valerobetaine, which increases the risk of obesity.^25^
- A host of chronic environmental exposures have been increasing since the 1960s, and Li and colleagues^26^ proposed that microplastics exposure in particular may be a driver for the observed changes in the age of onset of particular colon cancer types. This trend is shown in Figure 2. These potential exposures through ingestion of bottled foods and drinks, utensils, and other routes are suspected to affect the protective gastrointestinal mucosal layer, leading to changes in microbiota and other physiologic activities. Mechanistic and preclinical studies are needed to study further the chronic consumption of microplastics and CRC risk.
- Network science,^22^^,^^27^ computational pipelines,^28^ and artificial intelligence (AI) tools^29^ can organize data on environmental exposures and their interactions with biological responses. For example, specific chemical exposures during pregnancy have been linked to both dysregulated amino acids and early-onset breast cancer.^30^^,^^31^ Likewise, as new risk factors emerge for EOGIC,^26^^,^^32^ network science offers a powerful approach to linking multiple data entities, including those of exogenous and endogenous origins.

## Role of the microbiome

Evidence suggests that the human microbiome plays a role in cancer susceptibility. In particular, the possible roles of *Fusobacterium, Escherichia coli* strains that carry the polyketide synthase pathogenicity island *(pks+ E. coli), Enterotoxigenic B fragilis,* and other known microbes in GI cancers are well studied (Table 1), and colorectal cancer’s microbial environment was recently comprehensively reviewed.^35^ In 2012, 2 patient studies demonstrated for the first time the dominance of *Fusobacteria* in CRC tissue compared with the normal colon.^22^^,^^23^ Subsequent work supported these initial findings and further indicated the possible contribution of *Fusobacterium nucleatum* to metastasis and therapy response,^36^^,^^37^ as well as autophagy and chemoresistance.^38^ Mechanistic studies began to uncover its role as a promoter through its strong ability to form biofilms, which bind to and invade colonic epithelia and immune cells and induce protumorigenic signals.^39-42^

**Table 1.** Summary of key microbial species implicated in cancer development and their mechanisms of action.

Microbe	Cancer type	Mechanism
*Salmonella enterica*	Gallbladder	T3SS-dependent activation of PI3K/AKT and MAPK signaling, epigenetic modifications, AvrA-dependent β-catenin activation
*Fusobacterium nucleatum*	Colorectal, esophageal, oral squamous cell	β-Catenin-mediated activation of inflammatory and mitogenic transcriptional programs, EMT signaling
*Enterotoxigenic Bacteroides fragilis*	Colorectal	β-Catenin activation, MAPK signaling activation, E-cadherin cleavage
*Escherichia coli pks+*	Colorectal	Colibactin toxin induction of DNA damage
*Clostridium difficile*	Colorectal	*C difficile* toxin TcdB induction of Wnt signaling, reactive oxygen species, and protumorigenic mucosal immune responses (mice)
*Helicobacter pylori*	Colorectal, pancreatic, gastric, esophageal	VacA and CagA toxins cause DNA damage and reactive oxygen species and activate prosurvival and proliferation signals

Pathogenic bacteria, including *S enterica* and *F nucleatum,* activate host cell signaling pathways such as PI3K/AKT and β-catenin to drive oncogenesis. *E B fragilis* influences colorectal cancer through β-catenin and MAPK pathway activation, whereas *E coli* and *C difficile* induce DNA damage and inflammation through colibactin and TcdB toxins, respectively. The well-known carcinogen *H pylori* promotes DNA damage, immune evasion, and cell proliferation through the actions of VacA and other toxins, contributing to gastrointestinal cancers. Together, these bacterial mechanisms highlight the importance of microbial interactions in the initiation and progression of cancer.^33^

pks+ *E coli* encodes the genotoxin colibactin and can induce a specific mutational signature (SBS88-*pks*, ID18-*pks*) in CRC, which in turn generates inactivating mutations in the *APC* tumor suppressor gene.^43^ This mutational signature, present in approximately 10% of patients with CRC and colibactin-induced mutations, correlates with earlier onset CRC.^43^

Studies have also highlighted the significance of metagenomics, metabolomic, and multikingdom microbiota analyses in identifying bacterial and fungal biomarkers of CRC for early detection.^44^^,^^45^ These findings underscore the potential of the microbiome as a diagnostic tool and therapeutic target for GI cancers.

Think Tank participants had several recommendations regarding future efforts to evaluate the role of the microbiome in EOGIC as follows:

- To take advantage of multiomic approaches for the identification of microbial, metabolite, and proteomics data, standardization and expansion of these databases must be top priorities. Standardization applies to sampling procedures (eg, stool samples and mucosal biopsies from different niches in the gastrointestinal tract), the timing of sample collection (eg, before bowl preparation and preoperative antibiotics), and the inclusion of critical metadata (eg, diet and drugs). Such standards will be required for accurate comparison of findings across studies.
- An important focus should continue to be on mechanistic studies of microbes or metabolites that show promise as therapeutic targets. Recent large cohort studies in melanoma have demonstrated a critical association between the gut microbiome composition at baseline and response to immunotherapy.^46-49^ Critically, they monitored dietary and supplement/drug intake, which uncovered a key relationship between dietary fiber intake, the gut microbiota composition, and response to therapy. They used fecal microbiota transplantation to identify the dietary fibers that modified the microbiome to trigger an enhanced immune response.
- In addition, studies of clinical isolates of *F nucleatum* demonstrated that these isolates could propagate in metastatic cells through alteration of motility and extravasation signals.^50^ As such, blocking these signals or targeting these microbes for elimination could be tested as a means of preventing metastatic recurrence.
- Advances in strain-level and virulence-level detection, and consistency among experimental strains and taxonomic classification, are needed. Emerging evidence suggests that even among *F nucleatum*, it is the subspecies *F nucleatum animalis* Clade II (Fna C2) that is primarily driving the association between this species and CRC.^51^
- Continued innovation in ex vivo, in vitro, and animal models for elucidation of microbial tumorigenic mechanisms and therapeutic testing thereof are needed. Promising advances in models include organoids derived from human tissues, for which cocultures with immune cells and other cell types are now feasible, and “gut-on-a-chip” models that can replicate fluid dynamics and balance the an/aerobic needs of the gut microbiota/host epithelium. These models may advance personalized medicine, as patient-derived tumor biopsies could be tested in vitro for chemotherapeutic sensitivity while simultaneously also examining potential avenues for microbial interventions. Novel methodologies incorporating the microbiome into spatial transcriptomics and other modalities are also poised to rapidly expand our knowledge of how specific microbes contribute to intratumoral heterogeneity.^50^
- Circulating blood-based microbiome studies appear promising for research purposes but are in the nascent stages and will require rigorous testing before they can be useful clinically.
- Last, to accurately capture and identify the most salient factors associated with EOGIC, collaborations need to be established across the globe (eg, PROSPECT and OPTIMISTICC [https://www.cancergrandchallenges.org/prospect; https://www.cancergrandchallenges.org/optimisticc]). With the power of AI-informed models, these large globally representative datasets could be leveraged to reveal critical associations and patterns previously unrecognized. This information could lead to additional studies, both population level and mechanistic, that may result in preventative measures or early-detection tools for EOGICs.

## Survivorship

Survivorship is the domain of cancer care and research focused on overall health and well-being, which includes the physical, emotional, social, and financial aspects of cancer. Survivorship is especially relevant to long-term survivors of EOGIC due to the disproportionate burden of adverse impacts affecting younger patients facing decades of additional life. Further, patients treated for EOGIC are in the uniquely dynamic and challenging life stage of older adolescence and young adulthood (AYA; defined by the National Cancer Institute [NCI] as 15-39 years of age), which involves numerous interpersonal, educational, vocational, and financial challenges.^52^ Undoubtedly, most of what is true for AYAs in this regard applies to patients with EOGIC in their forties.^53^

### Late effects and health outcomes

“Late effects” refers to adverse physical or psychosocial impacts of cancer or its treatment that develop or persist following completion of treatment with curative intent. The increasing burden of late effects among EOGIC survivors is related to the rising incidence of sporadic EOGIC coupled with decreasing mortality in recent decades.^54^^,^^55^ Additionally, late effects are especially common in this younger population due to presentation with nonspecific symptoms, delayed diagnosis, more advanced stage, distal/rectal location of colorectal tumors, and a higher historical likelihood of more aggressive management using multimodal therapy.^54^^,^^56^ Local control measures consisting of surgical resection and/or irradiation may result in changes in body image; need for ostomy; chronic abdominopelvic pain; permanent alterations in urinary and bowel functions often manifesting as diarrhea, stool leakage, urinary incontinence, and infertility; and both male and female sexual dysfunction.^57-59^ Commonly used types of chemotherapy, including oxaliplatin, may result in persistent sensory neuropathy.^58-60^ The effects of cancer treatment are also thought to result in sarcopenia and accelerated aging.^61^^,^^62^

Newer strategies for reducing the above problems include limiting or even omitting aggressive surgical resections using minimally invasive laparoscopy and robotic platforms,^63^^,^^64^ as well as routinely using image-guided and intensity-modulated radiation therapy for more precise treatment with less off-target effects, especially with distal rectal tumors.^65^ The introduction of novel agents for the treatment of EOGIC, such as tyrosine kinase, BRAF, and PD-1/PDL-1 inhibitors, offers the potential for less reliance on platinum-based therapy, thus minimizing the effects of neuropathy, for example.^64^^,^^66^

A more comprehensive understanding of late effects following EOGIC could be achieved through the systematic, longitudinal study of adequately sized EOGIC patient cohorts. To our knowledge, no such cohorts currently exist but could be modeled after enormously productive pediatric cancer survivorship cohorts such as the Childhood Cancer Survivor Study developed in the early 1990s,^67^ as well as the Platinum Cohort^68^ focused on platinum toxicity in young adult males, and the Horizon^69^ and SURVAYA^70^ cohorts, both AYA-specific survivorship initiatives. An existing potential resource for EOGIC cohort-building is the NCI National Clinical Trials Network and the NCI Community Oncology Research Program, which together promote collaboration across NCI-funded cooperative oncology groups while increasing access to community-based practices, where most young adults receive cancer care.^71^^,^^72^ Expanded use of these and similar approaches is needed to address knowledge gaps, including (1) the types, prevalence, clinical trajectory, functional impact, and risk factors for known late effects and those emerging with novel agents; (2) the ability to risk-stratify patients through their genetic susceptibility; and (3) more individualized treatment planning that balances short- and long-term considerations, including patient preferences.

### Psychosocial impacts

Due to their diverse developmental stages, patients with EOGIC experience significant and unique interpersonal challenges that can affect their emotional well-being, including social isolation, body image, intimacy, and sexual and reproductive health.^73^^,^^74^ Compared with children and older adults or healthy peers, AYAs with cancer have higher levels of clinically significant distress, anxiety, and depression.^75-78^ This population also experiences more severe and longer lasting financial toxicity due to copays and other unreimbursed out-of-pocket expenses combined with a lack of accumulated wealth and financial resources, incomplete postsecondary education or vocational preparation, inability to return or start work due to illness and treatment demands, uncertain relationship status and potential lack of partner income, and possibly children and other dependents.^79-81^

In the future, optimal psychosocial care of patients with EOGICs requires (1) more research focused on this population, including those with intersectional identities encompassing race, ethnicity, gender, sexual orientation, and other characteristics associated with cancer disparities^82-84^; (2) expanded access to effective psychosocial support interventions in the community setting^85^; and (3) more complete understanding of their long-term cancer survivorship needs and multilevel strategies (patient, support system, institution, and health-care system) needed.^86^

### Survivorship care and services

Beginning in the early 1990s, the discipline of pediatric cancer survivorship emerged through the study of large patient cohorts and the introduction of risk-adapted monitoring standards for childhood cancer survivors, such as the Children’s Oncology Group Long-Term Follow-Up Guidelines for Survivors of Childhood, Adolescent, and Young Adult Cancer^67^ (https://survivorshipguidelines.org). Similar guidelines have now been developed for survivors of AYA/early-onset cancer by the National Comprehensive Cancer Network (https://nccn.org/professionals/physician_gls/pdf/aya.pdf). Such guidelines have contributed immeasurably to standardizing care and create the opportunity for modification based on future research evaluating their yield and value.

To our knowledge, there are no existing survivorship guidelines specific to the EOGIC population. Current GI cancer survivorship guidelines are exemplified by those of the American Cancer Society^59^ and the American Society of Colon and Rectal Surgeons.^87^ While addressing some of the previously mentioned late effects, they do not necessarily address all issues relevant to young adults, such as sexual health, infertility, and characteristic psychosocial challenges related to life stage. Until more EOGIC cohort data can be generated, survivorship clinicians must rely on a combination of disease-specific and AYA-focused survivorship recommendations to offer comprehensive survivorship care.^88-90^ Worth emphasizing is the value that health promotion and management of common comorbidities, such as obesity, poor diet, and inactivity, can have on cancer survivorship outcomes in younger populations.^91^^,^^92^

Think Tank recommendations included the following:

- Development and evaluation of survivorship care models (best practices) tailored to the young adult population.
- Expanded research into the psychosocial impacts of EOGIC, especially financial toxicity, overlapping disparities, and improving access to psychosocial services in community settings.
- Creation of supportive rehabilitative interventions aimed at improving cancer-related outcomes and quality of life in the younger population, such as pelvic floor rehabilitation, sacral nerve stimulation, dietary modifications, sexual health and therapy, and psychotherapy.
- Lifestyle modifications to optimize long-term health.
- Survivorship care for EOGIC patients achieving prolonged survival with chronic cancer therapy.
- Implementation of equitable survivorship care.

## Summary/conclusions

The EOGIC Think Tank was convened by the Ruesch Center for the Cure of Gastrointestinal Cancers, bringing together a diverse panel of experts in the fields of oncology, gastroenterology, epidemiology, translational and microbiome research, computational biology, and cancer survivorship, among others, to develop a roadmap for studying and tackling the EOGIC epidemic. The panel acknowledged the alarming rise of EOGICs, the associated racial and ethnic disparities, as well as the unique clinical and survivorship needs of EOGIC patients. Despite the possibility for some germline and environment interactions, the epidemiology of EOGICs and the associated birth cohort effect shine the light on the role of the exposome, especially that of early life, in EOGIC pathogenesis. To effectively study exposures and their biological relevance in cancer incidence, biology, and outcomes, the panel strongly advocated for the conduct of longitudinal early-life prospective and observational cohorts with matched lifestyle and biospecimen data. Novel technologies, such as mass-spectrometry, metabolomics, and generative AI tools, can be leveraged. However, international collaboration, expansion of the current databases, and standardization of measurements and analyses are also necessary. During the Think Tank, particular emphasis was given to the proposed role and accumulating evidence implicating the microbiome in EOGIC pathogenesis. Through direct genotoxicity, activation of oncogenic pathways, and modulation of the tumor-immune microenvironment, “oncomicrobes” such as *F nucleatum* and pks+ *E coli* have been the focus of extensive studies. However, when it comes to the microbiome, EOGIC researchers have to account for technical, interindividual, intraindividual, and bacterial strain variability in addition to pursuing mechanistic studies in faithful preclinical models to move past association to causation and, ultimately, clinical intervention. Another area of particular focus of the Think Tank was that of EOGIC survivorship and the disproportional and unique psychosocial challenges and late effects that patients with EOGIC face, which implore the development and equitable implementation of survivorship care models specifically tailored for this population. We hope that clinicians, researchers, patient advocates, and society will answer this call to action toward a better understanding of the biology of EOGICs, translating discovery into novel prevention, treatment, and survivorship paradigms and ultimately improving health outcomes for EOGIC patients.

## Data Availability

The authors commit to making all related data publicly available.

## References

[1] Ben-Aharon I , Laarhoven H, Fontana E, et al Early-onset cancer in the gastrointestinal tract is on the rise-evidence and implications. Cancer Discov. 2023;13:538-551. 36757194 10.1158/2159-8290.CD-22-1038

[2] Mauri G , Patelli G, Sartore-Bianchi A, et al Early-onset cancers: biological bases and clinical implications. Cell Rep Med. 2024;5:101737. 39260369 10.1016/j.xcrm.2024.101737 PMC11525030

[3] Surveillance, Epidemiology, and End Results (SEER) Program. SEERStat Database: incidence—SEER Research Plus Data, 12 Registries, Nov 2023 Sub (1992-2021)—linked to county attributes—total U.S., 1969-2022 counties, National Cancer Institute, DCCPS, Surveillance Research Program, released April 2024, based on the November 2023 submission. 2024. www.seer.cancer.gov

[4] Rich NE , Yopp AC, Singal AG, et al Hepatocellular carcinoma incidence is decreasing among younger adults in the United States. Clin Gastroenterol Hepatol. 2020;18:242-248.e5. 31042582 10.1016/j.cgh.2019.04.043 PMC6817412

[5] Bhutada JKS , Hwang AE, Liu L, Tsai K-Y, Deapen D, Freyer DR. Risk of presenting with poor-prognosis metastatic cancer in adolescents and young adults: a population-based study. Cancers. 2022;14:4932. 36230854 10.3390/cancers14194932 PMC9562204

[6] Sheth Bhutada J , Hwang A, Liu L, et al Poor-prognosis metastatic cancers in adolescents and young adults: incidence patterns, trends, and disparities. JNCI Cancer Spectr. 2021;5:pkab039. 10.1093/jncics/pkab039 PMC8266435 34250441

[7] Nieto Dominguez AJ , Eichinger SE, Guifarro D, et al Modifiable risk factors in Hispanic adults with gastric cancer in the United States. Cureus. 2024;16:e61920. 38978891 10.7759/cureus.61920 PMC11228387

[8] Grad YH , Lipsitch M, Aiello AE. Secular trends in *Helicobacter pylori* seroprevalence in adults in the United States: evidence for sustained race/ethnic disparities. Am J Epidemiol. 2012;175:54-59. 22085628 10.1093/aje/kwr288 PMC3244610

[9] Cheng YJ , Kanaya AM, Araneta MRG, et al Prevalence of diabetes by race and ethnicity in the United States, 2011-2016. JAMA. 2019;322:2389-2398. 31860047 10.1001/jama.2019.19365 PMC6990660

[10] Ogino S , Ugai T. The global epidemic of early-onset cancer: nature, nurture, or both? Ann Oncol. 2024;35:1071-1073. 10.1016/j.annonc.2024.08.2336 39293513 PMC11624085

[11] Rosenberg PS , Miranda-Filho A. Cancer incidence trends in successive social generations in the US. JAMA Netw Open. 2024;7:e2415731. 38857048 10.1001/jamanetworkopen.2024.15731 PMC11165384

[12] Sung H , Jiang C, Bandi P, et al Differences in cancer rates among adults born between 1920 and 1990 in the USA: an analysis of population-based cancer registry data. Lancet Public Health. 2024;9:e583-e593. 39095135 10.1016/S2468-2667(24)00156-7

[13] Gupta S , May FP, Kupfer SS, et al Birth cohort colorectal cancer (CRC): implications for research and practice. Clin Gastroenterol Hepatol. 2024;22:455-469.e7. 38081492 10.1016/j.cgh.2023.11.040 PMC11304405

[14] Pearlman R , Frankel WL, Swanson B, et al; Ohio Colorectal Cancer Prevention Initiative Study Group. Prevalence and spectrum of germline cancer susceptibility gene mutations among patients with early-onset colorectal cancer. JAMA Oncol. 2017;3:464-471. 27978560 10.1001/jamaoncol.2016.5194 PMC5564179

[15] Stoffel EM , Koeppe E, Everett J, et al Germline genetic features of young individuals with colorectal cancer. Gastroenterology. 2018;154:897-905.e1. 29146522 10.1053/j.gastro.2017.11.004 PMC5847426

[16] You YN , Moskowitz JB, Chang GJ, et al Germline cancer risk profiles of patients with young-onset colorectal cancer: findings from a prospective universal germline testing and telegenetics program. Dis Colon Rectum. 2023;66:531-542. 35195555 10.1097/DCR.0000000000002347

[17] Vogelaar IP , van der Post RS, van Krieken JHJ, et al Unraveling genetic predisposition to familial or early onset gastric cancer using germline whole-exome sequencing. Eur J Hum Genet. 2017;25:1246-1252. 28875981 10.1038/ejhg.2017.138 PMC5643972

[18] Salo-Mullen EE , O’Reilly EM, Kelsen DP, et al Identification of germline genetic mutations in patients with pancreatic cancer. Cancer. 2015;121:4382-4388. 26440929 10.1002/cncr.29664 PMC5193099

[19] Archambault AN , Su YR, Jeon J, et al Cumulative burden of colorectal cancer-associated genetic variants is more strongly associated with early-onset vs late-onset cancer. Gastroenterology. 2020;158:1274-1286.e12. 31866242 10.1053/j.gastro.2019.12.012 PMC7103489

[20] Laskar RS , Qu C, Huyghe JR, et al; Colorectal Transdisciplinary (CORECT) Study, the Colon Cancer Family Registry (CCFR), Genetics and Epidemiology of Colorectal Cancer Consortium (GECCO). Genome-wide association studies and Mendelian randomization analyses provide insights into the causes of early-onset colorectal cancer. Ann Oncol. 2024;35:523-536. 38408508 10.1016/j.annonc.2024.02.008 PMC11213623

[21] Miller GW , Jones DP. The nature of nurture: refining the definition of the exposome. Toxicol Sci. 2014;137:1-2. 24213143 10.1093/toxsci/kft251 PMC3871934

[22] Vermeulen R , Schymanski EL, Barabasi AL, et al The exposome and health: where chemistry meets biology. Science. 2020;367:392-396. 31974245 10.1126/science.aay3164 PMC7227413

[23] Wild CP. Complementing the genome with an “exposome”: the outstanding challenge of environmental exposure measurement in molecular epidemiology. Cancer Epidemiol Biomarkers Prev. 2005;14:1847-1850. 16103423 10.1158/1055-9965.EPI-05-0456

[24] Pronk A , Loh M, Kuijpers E, et al; EPHOR Consortium. Applying the exposome concept to working life health: the EU EPHOR project. Environ Epidemiol. 2022;6:e185. 35434456 10.1097/EE9.0000000000000185 PMC9005258

[25] Liu KH , Owens JA, Saeedi B, et al Microbial metabolite delta-valerobetaine is a diet-dependent obesogen. Nat Metab. 2021;3:1694-1705. 34931082 10.1038/s42255-021-00502-8 PMC8711632

[26] Li S , Keenan JI, Shaw IC, Frizelle FA. Could microplastics be a driver for early onset colorectal cancer?. Cancers. 2023;15:3323. 37444433 10.3390/cancers15133323 PMC10340669

[27] Hu X , Go YM, Jones DP. Omics integration for mitochondria systems biology. Antioxid Redox Signal. 2020;32:853-872. 31891667 10.1089/ars.2019.8006 PMC7074923

[28] Uppal K , Walker DI, Liu K, et al Computational metabolomics: a framework for the million metabolome. Chem Res Toxicol. 2016;29:1956-1975. 27629808 10.1021/acs.chemrestox.6b00179 PMC5376098

[29] Merino Martinez R , Muller H, Negru S, et al Human exposome assessment platform. Environ Epidemiol. 2021;5:e182. 34909561 10.1097/EE9.0000000000000182 PMC8663864

[30] Go YM , Weinberg J, Teeny S, et al Exposome epidemiology for suspect environmental chemical exposures during pregnancy linked to subsequent breast cancer diagnosis. Environ Int. 2023;178:108112. 37517180 10.1016/j.envint.2023.108112 PMC10863607

[31] Teeny S , Jarrell ZR, Krigbaum NY, et al Third trimester serum amino acid metabolism is associated with maternal breast cancer diagnosed within 15 years of pregnancy. Res Sq. 2023:rs.3.rs-3272893. 10.21203/rs.3.rs-3272893/v1

[32] Helte E , Save-Soderbergh M, Larsson SC, et al Disinfection by-products in drinking water and risk of colorectal cancer: a population-based cohort study. J Natl Cancer Inst. 2023;115:1597-1604. 37551954 10.1093/jnci/djad145 PMC10699800

[33] Geyer R. Annual plastic production between 1950 and 2019. 2023. https://ourworldindata.org/plastic-pollution

[34] Gagnaire A , Nadel B, Raoult D, et al Collateral damage: insights into bacterial mechanisms that predispose host cells to cancer. Nat Rev Microbiol. 2017;15:109-128. 28045107 10.1038/nrmicro.2016.171

[35] White MT , Sears CL. The microbial landscape of colorectal cancer. Nat Rev Microbiol. 2024;22:240-254. 37794172 10.1038/s41579-023-00973-4

[36] Castellarin M , Warren RL, Freeman JD, et al *Fusobacterium nucleatum* infection is prevalent in human colorectal carcinoma. Genome Res. 2012;22:299-306. 22009989 10.1101/gr.126516.111 PMC3266037

[37] Kostic AD , Gevers D, Pedamallu CS, et al Genomic analysis identifies association of Fusobacterium with colorectal carcinoma. Genome Res. 2012;22:292-298. 22009990 10.1101/gr.126573.111 PMC3266036

[38] Ramos A , Hemann MT. Drugs, bugs, and cancer: *Fusobacterium nucleatum* promotes chemoresistance in colorectal cancer. Cell. 2017;170:411-413. 28753421 10.1016/j.cell.2017.07.018

[39] Bullman S , Pedamallu CS, Sicinska E, et al Analysis of Fusobacterium persistence and antibiotic response in colorectal cancer. Science. 2017;358:1443-1448. 29170280 10.1126/science.aal5240 PMC5823247

[40] Casasanta MA , Yoo CC, Udayasuryan B, et al *Fusobacterium nucleatum* host-cell binding and invasion induces IL-8 and CXCL1 secretion that drives colorectal cancer cell migration. Sci Signal. 2020;13:eaba9157. 10.1126/scisignal.aba9157 PMC7454160 32694172

[41] Mima K , Sukawa Y, Nishihara R, et al *Fusobacterium nucleatum* and T cells in colorectal carcinoma. JAMA Oncol. 2015;1:653-661. 26181352 10.1001/jamaoncol.2015.1377 PMC4537376

[42] Ye X , Wang R, Bhattacharya R, et al *Fusobacterium nucleatum* subspecies animalis influences proinflammatory cytokine expression and monocyte activation in human colorectal tumors. Cancer Prev Res (Phila). 2017;10:398-409. 28483840 10.1158/1940-6207.CAPR-16-0178

[43] Rosendahl Huber A , Pleguezuelos-Manzano C, Puschhof J, et al Improved detection of colibactin-induced mutations by genotoxic *E. coli* in organoids and colorectal cancer. Cancer Cell. 2024;42:487-496.e6. 38471458 10.1016/j.ccell.2024.02.009

[44] Dai Z , Coker OO, Nakatsu G, et al Multi-cohort analysis of colorectal cancer metagenome identified altered bacteria across populations and universal bacterial markers. Microbiome. 2018;6:70. 29642940 10.1186/s40168-018-0451-2 PMC5896039

[45] Liu NN , Jiao N, Tan JC, et al Multi-kingdom microbiota analyses identify bacterial-fungal interactions and biomarkers of colorectal cancer across cohorts. Nat Microbiol. 2022;7:238-250. 35087227 10.1038/s41564-021-01030-7 PMC8813618

[46] Baruch EN , Youngster I, Ben-Betzalel G, et al Fecal microbiota transplant promotes response in immunotherapy-refractory melanoma patients. Science. 2021;371:602-609. 33303685 10.1126/science.abb5920

[47] Lee KA , Thomas AM, Bolte LA, et al Cross-cohort gut microbiome associations with immune checkpoint inhibitor response in advanced melanoma. Nat Med. 2022;28:535-544. 35228751 10.1038/s41591-022-01695-5 PMC8938272

[48] Simpson RC , Shanahan ER, Batten M, et al Diet-driven microbial ecology underpins associations between cancer immunotherapy outcomes and the gut microbiome. Nat Med. 2022;28:2344-2352. 36138151 10.1038/s41591-022-01965-2

[49] Williams N , Wheeler CE, Husain M, et al De-correlating immune checkpoint inhibitor toxicity and response in melanoma via the microbiome. J Clin Oncol. 2023;41:9569.

[50] Galeano Nino JL , Wu H, LaCourse KD, et al Effect of the intratumoral microbiota on spatial and cellular heterogeneity in cancer. Nature. 2022;611:810-817. 36385528 10.1038/s41586-022-05435-0 PMC9684076

[51] Zepeda-Rivera M , Minot SS, Bouzek H, et al A distinct *Fusobacterium nucleatum* clade dominates the colorectal cancer niche. Nature. 2024;628:424-432. 38509359 10.1038/s41586-024-07182-w PMC11006615

[52] Overholser L , Kilbourn K, Liu A. Survivorship issues in adolescent and young adult oncology. Med Clin North Am. 2017;101:1075-1084. 28992855 10.1016/j.mcna.2017.06.002

[53] Eng C , Jacome AA, Agarwal R, et al A comprehensive framework for early-onset colorectal cancer research. Lancet Oncol. 2022;23:e116-e128. 35090673 10.1016/S1470-2045(21)00588-X

[54] Harrold E , Latham A, Pemmaraju N, et al Early-onset GI cancers: rising trends, genetic risks, novel strategies, and special considerations. Am Soc Clin Oncol Educ Book. 2023;43:e398068. 10.1200/edbk_398068(43):e398068 37235819

[55] Lewis DR , Siembida EJ, Seibel NL, et al Survival outcomes for cancer types with the highest death rates for adolescents and young adults, 1975-2016. Cancer. 2021;127:4277-4286. 34308557 10.1002/cncr.33793

[56] Christodoulides N , Lami M, Malietzis G, et al Sporadic colorectal cancer in adolescents and young adults: a scoping review of a growing healthcare concern. Int J Colorectal Dis. 2020;35:1413-1421. 32556652 10.1007/s00384-020-03660-5 PMC7340664

[57] Buccafusca G , Proserpio I, Tralongo AC, et al Early colorectal cancer: diagnosis, treatment and survivorship care. Crit Rev Oncol Hematol. 2019;136:20-30. 30878125 10.1016/j.critrevonc.2019.01.023

[58] Denlinger CS , Barsevick AM. The challenges of colorectal cancer survivorship. J Natl Compr Canc Netw. 2009;7:883-893; quiz 894. 19755048 10.6004/jnccn.2009.0058 PMC3110673

[59] El-Shami K , Oeffinger KC, Erb NL, et al American Cancer Society Colorectal Cancer Survivorship Care guidelines. CA Cancer J Clin. 2015;65:428-455. 26348643 10.3322/caac.21286 PMC5385892

[60] Kang L , Tian Y, Xu S, et al Oxaliplatin-induced peripheral neuropathy: clinical features, mechanisms, prevention and treatment. J Neurol. 2021;268:3269-3282. 32474658 10.1007/s00415-020-09942-w

[61] Guida JL , Hyun G, Belsky DW, et al Associations of seven measures of biological age acceleration with frailty and all-cause mortality among adult survivors of childhood cancer in the St Jude Lifetime Cohort. Nat Cancer. 2024;5:731-741. 38553617 10.1038/s43018-024-00745-w PMC11139608

[62] Wang S , El Jurdi N, Thyagarajan B, Prizment A, Blaes AH. Accelerated aging in cancer survivors: cellular senescence, frailty, and possible opportunities for interventions. IJMS. 2024;25:3319. 38542292 10.3390/ijms25063319 PMC10970400

[63] Sheng S , Zhao T, Wang X. Comparison of robot-assisted surgery, laparoscopic-assisted surgery, and open surgery for the treatment of colorectal cancer: a network meta-analysis. Medicine (Baltimore). 2018;97:e11817. 30142771 10.1097/MD.0000000000011817 PMC6112974

[64] Cercek A , Sinopoli JC, Shia J, et al Durable complete responses to PD-1 blockade alone in mismatch repair deficient locally advanced rectal cancer. J Clin Oncol. 2024;42:LBA3512.

[65] Damico N , Meyer J, Das P, et al ECOG-ACRIN guideline for contouring and treatment of early stage anal cancer using IMRT/IGRT. Pract Radiat Oncol. 2022;12:335-347. 35717050 10.1016/j.prro.2022.01.015

[66] Nalli M , Puxeddu M, La Regina G, et al Emerging therapeutic agents for colorectal cancer. Molecules. 2021;26:7463. 34946546 10.3390/molecules26247463 PMC8707340

[67] Robison LL , Armstrong GT, Boice JD, et al The childhood cancer survivor study: a National Cancer Institute-supported resource for outcome and intervention research. J Clin Oncol. 2009;27:2308-2318. 19364948 10.1200/JCO.2009.22.3339 PMC2677920

[68] Sanchez VA , Shuey MM, Dinh PC Jr, et al Patient-reported functional impairment due to hearing loss and tinnitus after cisplatin-based chemotherapy. J Clin Oncol. 2023;41:2211-2226. 36626694 10.1200/JCO.22.01456 PMC10489421

[69] Nichols HB , Baggett CD, Engel SM, et al The adolescent and young adult (AYA) horizon study: an AYA cancer survivorship cohort. Cancer Epidemiol Biomarkers Prev. 2021;30:857-866. 33619021 10.1158/1055-9965.EPI-20-1315 PMC8102328

[70] Vlooswijk C , Poll-Franse LVV, Janssen SHM, et al Recruiting adolescent and young adult cancer survivors for patient-reported outcome research: experiences and sample characteristics of the SURVAYA study. Curr Oncol. 2022;29:5407-5425. 36005166 10.3390/curroncol29080428 PMC9406992

[71] Roth ME , Grimes AC, Reed DR, et al Children’s Oncology Group 2023 blueprint for research: adolescent and young adult oncology. Pediatr Blood Cancer. 2023;70(Suppl 6):e30564. 37439574 10.1002/pbc.30564 PMC10630986

[72] Weiss AR , Nichols CR, Freyer DR. Enhancing adolescent and young adult oncology research within the National Clinical Trials Network: rationale, progress, and emerging strategies. Semin Oncol. 2015;42:740-747. 26433555 10.1053/j.seminoncol.2015.07.012 PMC4604069

[73] Fladeboe KM , Siembida EJ, Ip E, et al Indicators of developmental status among adolescents and young adults with cancer: perceived adult status, social milestones, and health-related quality of life. Psychooncology. 2023;32:1363-1371. 37381114 10.1002/pon.6186 PMC11801360

[74] Arem H , Duarte DA, White B, et al Young adult cancer survivors’ perspectives on cancer’s impact on different life areas post-treatment: a qualitative study. J Adolesc Young Adult Oncol. 2024;13:748-759. 10.1089/jayao.2024.0021 38695773 PMC11564678

[75] Kaul S , Avila JC, Mutambudzi M, et al Mental distress and health care use among survivors of adolescent and young adult cancer: a cross-sectional analysis of the National Health Interview Survey. Cancer. 2017;123:869-878. 27859009 10.1002/cncr.30417

[76] McCarthy MC , McNeil R, Drew S, et al Psychological distress and posttraumatic stress symptoms in adolescents and young adults with cancer and their parents. J Adolesc Young Adult Oncol. 2016;5:322-329. 27214245 10.1089/jayao.2016.0015

[77] Sansom-Daly UM , Wakefield CE. Distress and adjustment among adolescents and young adults with cancer: an empirical and conceptual review. Transl Pediatr. 2013;2:167-197. 26835313 10.3978/j.issn.2224-4336.2013.10.06 PMC4729076

[78] Bailey CE , Tran Cao HS, Hu CY, et al Functional deficits and symptoms of long-term survivors of colorectal cancer treated by multimodality therapy differ by age at diagnosis. J Gastrointest Surg. 2015;19:180-188; discussion 188. 25213581 10.1007/s11605-014-2645-7 PMC4289079

[79] Berghuijs K , Kaddas HK, Trujillo G, et al Age-related differences in employment, insurance, and financial hardship among colorectal cancer patients: a report from the ColoCare Study. J Cancer Surviv. 2024;18:1075-1084. 36949233 10.1007/s11764-023-01362-9 PMC11827535

[80] Lu AD , Zheng Z, Han X, et al Medical financial hardship in survivors of adolescent and young adult cancer in the United States. J Natl Cancer Inst. 2021;113:997-1004. 33839786 10.1093/jnci/djab013 PMC8328985

[81] Parsons SK , Keegan THM, Kirchhoff AC, et al Cost of cancer in adolescents and young adults in the United States: results of the 2021 report by Deloitte Access Economics, commissioned by Teen Cancer America. J Clin Oncol. 2023;41:3260-3268. 36827624 10.1200/JCO.22.01985 PMC10256335

[82] Choi E , Berkman AM, Andersen CR, et al Psychological distress and mental health care utilization among lesbian, gay, and bisexual survivors of adolescent and young adult cancer. Support Care Cancer. 2024;32:585. 39134915 10.1007/s00520-024-08778-8

[83] Choi E , Berkman AM, Battle A, et al Psychological distress and mental health care utilization among Black survivors of adolescent and young adult cancer. Cancer. 2024;130:3011-3022. 38676935 10.1002/cncr.35348 PMC11309887

[84] Choi E , Berkman AM, Cheung CK, et al Psychological distress and mental health care utilization among Hispanic/Latino survivors of adolescent and young adult cancer. Psychooncology. 2023;32:1918-1929. 37955581 10.1002/pon.6248 PMC10872722

[85] Beauchemin MP , Ji L, Williams AM, et al Defining practice capacity for cancer care delivery to adolescents and young adults in the community setting: 2022 landscape assessment results. J Adolesc Young Adult Oncol. 2024;13:557-563. 38394227 10.1089/jayao.2023.0177 PMC11296314

[86] McGrady ME , Willard VW, Williams AM, et al Psychological outcomes in adolescent and young adult cancer survivors. J Clin Oncol. 2024;42:707-716. 37967297 10.1200/JCO.23.01465 PMC13019686

[87] Hardiman KM , Felder SI, Friedman G, et al; Prepared on behalf of the Clinical Practice Guidelines Committee of the American Society of Colon and Rectal Surgeons. The American Society of Colon and Rectal Surgeons Clinical Practice guidelines for the surveillance and survivorship care of patients after curative treatment of colon and rectal cancer. Dis Colon Rectum. 2021;64:517-533. 33591043 10.1097/DCR.0000000000001984

[88] Berkman AM , Betts AC, Beauchemin M, et al Survivorship after adolescent and young adult cancer: models of care, disparities, and opportunities. J Natl Cancer Inst. 2024;116:1417-1428. 10.1093/jnci/djae119 38833671 PMC11378318

[89] Haas S , Mikkelsen AH, Kronborg CJS, et al Management of treatment-related sequelae following colorectal cancer. Colorectal Dis. 2023;25:458-488. 35969031 10.1111/codi.16299

[90] Hutchinson T , Hoffe S, Saeed S, et al Gastrointestinal disease-specific survivorship care: a new personalized model integrating onco-wellness. Cancer Control. 2021;28:10732748211006081. 33926264 10.1177/10732748211006081 PMC8204645

[91] Dixon SB , Liu Q, Chow EJ, et al Specific causes of excess late mortality and association with modifiable risk factors among survivors of childhood cancer: a report from the Childhood Cancer Survivor Study cohort. Lancet. 2023;401:1447-1457. 37030315 10.1016/S0140-6736(22)02471-0 PMC10149583

[92] Tonorezos ES , Marcil V. Childhood cancer survivors: healthy behaviours and late mortality. Lancet. 2023;401:1403-1405. 37030313 10.1016/S0140-6736(22)02632-0

